# EU Regulatory Risk Management of Marine Biotoxins in the Marine Bivalve Mollusc Food-Chain

**DOI:** 10.3390/toxins10030118

**Published:** 2018-03-10

**Authors:** Micheál O’Mahony

**Affiliations:** Sea Fisheries Protection Authority, Clonakilty, Co. Cork P85 TX47, Ireland ; micheal.omahony@sfpa.ie

**Keywords:** biotoxin, phycotoxin, aquatic toxin, mollusc, food safety, risk management, regulation

## Abstract

Food safety risk assessment in the European Union (EU) recognises consumer illness that arises from marine biotoxins as a risk associated with bivalve mollusc consumption. EU food regulations contain various general food safety obligations, which should contribute significantly to managing this risk. EU food regulations additionally impose various specific obligations on both Food Business Operators and Competent Authorities in order to manage the marine biotoxin food safety risk in the bivalve mollusc food-chain. These have a particular focus on the pre-harvest component of the food-chain. A central component of these specific systems is the requirement for ongoing monitoring of phytoplankton and biotoxin concentrations in water and molluscs, respectively. This monitoring explicitly brings a potential outcome of closing production areas delineated by classification to prohibit the harvest of bivalve molluscs as food from those areas when acceptable biotoxin concentrations are exceeded. This review considers the utility of these systems, at conceptual and practical levels, and explores their contribution to an effective regulatory risk management approach.

## 1. Introduction

In EU Food regulations, ‘Marine Biotoxins’ means poisonous substances accumulated by bivalve molluscs, in particular as a result of feeding on plankton containing toxins [1] (Annex I, 2.2). Biotoxins are naturally produced by various species of marine phytoplankton, and may bio-accumulate in marine animals through normal physiological activity during their growing period in marine environment. Biotoxin accumulation in marine organisms subsequently consumed as food brings the consequent potential of reaching concentrations that create a toxicosis risk in consumers of those products following harvest. Consumer illness is most commonly acute gastroenteritis, with some toxin groups bringing the potential for more serious neurotoxic illness. In 2015 there were 44 outbreaks of biotoxin-related illness reported in EU consumers [2] (p. 199). However, current epidemiological systems have limited sensitivity when it comes to reliably detecting and recording illness that arises for marine biotoxins, with the resultant potential for significant underreporting of the actual public health burden [3] (p. 8). In addition to uncertainty around the incidence of illness arising from marine biotoxins, there is additional uncertainty in the attribution of illness to individual food products and food chains. Nevertheless, Bivalve Molluscs (BMs) are recognised as posing specific marine biotoxin food safety risks, and in the European Union, the food chain most frequently implicated in biotoxin-related foodborne disease is that of bivalve molluscs, with the occasional implication of other fishery products including those derived from crustaceans and fin-fish. 

The inherent biotoxin risk in the bivalve mollusc food chain arises from the normal filter-feeding biology of these animals, which creates the potential for the bioaccumulation of biotoxins in their tissues, with a consequent magnification of the environmental concentration of toxins. A further aspect of inherent risk with this food-chain is a consumption pattern by humans that frequently includes all soft-tissue parts of bivalve molluscs, particularly in the case of smaller mollusc species in which the removal of specific body-parts is physically impractical. Consumers therefore ingest organs and tissues involved in the physiological detoxification, sequestration, and excretion of chemical contaminants. Whilst such organs may also be consumed in other animal-origin food chains, selective consumption of limited types of tissues such as muscle (meat) is generally more prevalent than consumption of edible offal [4,5]. EU food safety regulations therefore set out regulatory requirements that are designed to contribute to the management of the inherent risk of this hazard group in the bivalve mollusc food chain. From a risk management perspective, the hazard enters the food-chain in the pre-harvest phase of the foodchain; therefore, the overt focus of risk management systems is on managing risk within that phase.

### EU Marine Bivalve Mollusc Production and Market

Bivalve mollusc production involves one of two broad approaches for deriving food products from these animals: either by capturing animals in wild fisheries or by farming animals to enhance production before harvest. Quantitative descriptions of the EU production and market of fishery products are collated and published by the Directorate—General for Maritime Affairs and Fisheries of the European Commission [6].

Data for 2015 estimate EU mollusc production at approximately 879,000 tonnes, with 629,000 tonnes (72%) farmed and the remaining 250,000 tonnes (28%) wild-caught. The main farmed species in the EU is mussels, followed by oysters and clams. The main wild-caught species landed in the EU is scallop, with a smaller contribution from mussels, oysters, and clams. In the present context, it is worth noting that harmful algal blooms with resultant biotoxin accumulation and consequent harvesting prohibition are recurrently referred to as limiting factor for EU mollusc production, due to on-going potential for closures of production areas.

In terms of the market, molluscs comprise approximately 11% of EU seafood consumption, with the EU being 65% self-sufficient in supplying internal mollusc demand and otherwise dependant on imported products. Mussels are the most consumed mollusc species by EU consumers, followed by scallops and then clams. 

## 2. EU Food Safety Regulatory Framework for Marine Biotoxins in Bivalve Molluscs

### 2.1. Overview & Terminology

In general terms, regulatory risk management of marine biotoxins in the bivalve mollusc food-chain is achieved in EU Food regulations through both general food safety obligations and specific biotoxin obligations. These two categories exist for both Food Business Operators (FBOs) and Competent Authorities (CAs), respectively. 

Food Business Operators is a regulatory term, which includes all of the participants in food production, including pre-harvest production, harvesting, and post-harvest processing, distribution and preparation of food. In the context of bivalve mollusc chain, the concept of FBO includes primary food producers such as farmers, gatherers, fishers, and initial transporters; also, operators of approved food establishments, such as dispatch centres, purification centres, and processors; as well as further distributors such as wholesalers, agents, and cold-store operators; and finally retailers such as supermarket, restaurant, and catering operators. In very general terms, FBOs are charged through food safety regulations by ensuring the food that they produce is safe, and they are additionally subject to specific obligations to manage specific risks in particular food groups.

Competent Authorities (CAs) are the public regulatory authorities tasked with ensuring compliance with such regulations in individual countries. Their roles are achieved through official controls, which include inspection audit sampling and analysis, to verify compliance. CA roles normally also potentially include enforcement of compliance where necessary. The role required of CAs in food legislation is primarily the verification of operator compliance with their obligations, but also includes requirements to establish specific official control systems to contribute to management of specific risks in particular food groups.

Bivalve molluscs may be consumed as live animals or dead entire animals, or in the case of larger molluscs consumption they may just involve limited body parts. EU legislation has mutually exclusive definitions of Live Bivalve Molluscs and Fishery Products, with the latter including processed whole or part animals derived from live bivalve molluscs. Thus the processing of live entire bivalve molluscs by typically cooking, freezing, or de-shelling or evisceration moves them from one legal category of live bivalve molluscs to a different legal category of fishery products. The legal concept of Live Bivalve Molluscs therefore broadly includes animals in the primary phase of food production prior to harvest as food, post-harvest animals not yet prepared as food, e.g., primary food products en route to a processing establishment or dispatch centre, as well as final ready-to-eat foods. The bivalve mollusc food chain can also involve processes such as cooking, freezing, or evisceration before consumption, with resultant consumption of entire dead animals or just specific parts (tissues or organs) of larger bivalve molluscs. 

### 2.2. General Regulatory Obligations for Marine Biotoxins: Food Business Operators

Regulations (EC) No. 178 of 2002 on general principles and requirements of food law (178/2002) [7], and (EC) No. 852 of 2004 on the hygiene of foodstuffs (852/2004) [8], create general obligations for all operators producing or placing food on the market in Europe. Within Regulation 178/2002, all Food Business Operators are required to ensure that they only place on the market food that is both safe and compliant with relevant requirements. Operators are obliged to have systems in place to both ensure and verify that the food they produce is compliant. If an operator finds that food has been dispatched that is not in compliance with relevant requirements, they are obliged to notify the CA and withdraw, and possibly recall, the product from the market. FBOs are generally required to maintain traceability of products received and dispatched, one-step forward and one step back, through supplier and customer details, with the notable exception of retailers who are not obliged to maintain recipient details. Imported foods are required to meet the same standards as indigenously produced food.

Regulation 852/2004 imposes further general hygiene obligations on all FBOs, broadly framed in two areas of facilities and systems. Primary food producers are obliged to comply with a specific Annex of 852/2004, which in the biotoxin context notably includes the general requirement to protect primary food products from contamination, and to take into account results of any analyses carried out on samples taken from animals that have importance to human health. All operators have general hygiene obligations for processes and facilities set out in various annexes of this regulation, and are generally obliged to appropriately adapt measures such as microbiological criteria temperature control, and sampling and analysis. All FBOs other than primary food producers are obliged to have food safety management procedures based upon ‘Hazard Analysis and Critical Control Point’ (HACCP) principles [9]. Thus, there should be active consideration by operators of what hazards are pertinent to their production systems, and attention should be given to the control of those at apposite points of the process, with on-going verification of the effectiveness of risk management. This regulation also provides for industry-led, sector-specific guides to good hygienic practice, which should help operators to identify practical, industry-led approaches to achieving the objective of the regulations. 

When bivalve molluscs are processed, e.g., by cooking, freezing, or evisceration, they become regarded as fishery products, and in addition to the above regulations those processes are subject to the provisions of a further regulation EC No 853 of 2004 that lays down specific hygiene rules for foods of animal origin (853/2004) [1]. This regulation has general applicability for operators other than primary food producers or retailers who handle or process foods of animal origin, including molluscs and their products. That regulation imposes general requirements for operators to ensure that fishery products are not placed on the market with biotoxin limits that are in excess of prescribed standards.

### 2.3. General Regulatory Obligations for Marine Biotoxins: Competent Authorities

Regulation 178/2002 creates a general obligation for Competent Authorities of EU Member States to verify and enforce compliance of all operators with their obligations. This regulation also obliges CAs to develop communication systems to deal with risk scenarios, including situations in which unsafe food has been placed on the market in different EU MS, namely the EU Rapid Alert System for Food and Feed Safety (RASFF). Regulation 852/2004 in conjunction with Regulation 853/2004 requires that certain food production establishments are approved by CAs for the food processes they undertake, prior to placing food on the market, whilst other establishments such as primary phase of food production, distribution, or retail establishments merely have to register with CAs. Approval is a regulatory concept that requires CAs to assess the adequacy of structures and systems to ensure safety of processing of foods of animal origin; only production in these establishments that meets the necessary standards is allowed. These three regulations require CAs to establish systems to ensure that imported products meet equivalent requirements of indigenous products.

The general organisation requirements for official controls performed by CAs are set out in Regulation (EC) No. 882 of 2004 on official controls performed to ensure verification of food safety and other laws (882/2004) [10]. It requires that official controls should be performed regularly, on a risk-basis, and with appropriate frequency. Official controls should include inspection audit sampling and analysis. In addition to this broad framework, Regulation (EC) No. 854 of 2004 laying down specific rules for the organisation of official controls on products of animal origin intended for human consumption (854/2004) [11] requires that CA official controls of establishments placing products of animal origin on the market should verify FBOs’ compliance with their particular requirements including hygiene and HACCP-based procedures. Such CA audits of FBO systems are required, in particular, to focus on systems designed to guarantee compliance of products with microbiological criteria, and legislation on residues, contaminants, and prohibited substances. Fishery products including fishery products derived from bivalve molluscs are subject to general requirements in Regulation 854/2004 for official checks to control contaminant levels and ensure that fishery products are not placed on the market with biotoxins in excess of relevant standards. CAs are required to declare such fishery products as unfit for human consumption. 

### 2.4. Specific Regulatory Obligations for Marine Biotoxins: Competent Authorities

In addition to the preceding general official control requirements, Regulation 854/2004 contains a dedicated Annex that sets out obligations for CAs to establish specific official control systems around Live Bivalve Mollusc production [11] (pp. 119–121). The overall focus is the creation of a purpose-designed official control system around the primary phase of bivalve mollusc production. These specific official control requirements are ostensibly designed to contribute to the management of food safety risks that arise from two broad hazard types on the basis that they pose particular risks in the bivalve mollusc food chain:–microbes arising from anthropogenic inputs, and the subject of this present review, namely, marine biotoxins arising from naturally occurring algal blooms.

A central tenet of this official control system is the regulatory concept of classification of BM production areas. Thus, the LBM annex of Regulation 854/2004 requires Competent Authorities to delineate the boundaries of production areas from which molluscs may be harvested and award a microbiological classification, one of A, B, or C according to sanitary survey and microbiological monitoring. The category of classification allocated by the CA is designed to be a general categorisation of inherent risks from microbiological and other anthropogenic inputs to the area. Therefore, the classification category does not in any way characterise the risk of bio-toxin accumulation in that area. However, the resultant, geographically-delineated classified production area is also the risk management unit of interest for marine biotoxins. In such classified production areas, competent authorities should establish monitoring programmes, based upon representative sampling and analysis to examine concentration of microbes, biotoxins, toxin-producing plankton, and other chemical contaminants. 

Those official control monitoring programmes should specifically check for toxin-producing phytoplankton in water and biotoxins in mollusc flesh. Phytoplankton monitoring is required to be representative of water column, and phytoplankton population trends indicating increased toxin accumulation risk should result in increased toxin monitoring. The sampling frequency for biotoxins is, as a general rule, to be weekly, but higher or lower frequencies may arise on the basis of risk assessment, which should be periodically reviewed. Within one area, official control biotoxin monitoring may rely on an ‘indicator species’ of mollusc known to be the highest biotoxin accumulator. Whilst the overt focus would implicitly seem to be around those biotoxins for which a health standard is prescribed in 853/2004, Competent Authority monitoring obligations are not limited to such toxins and also extend to requiring periodic monitoring for new and unknown toxins, as stipulated in Regulation 2074 of 2005 implementing certain measures of various regulations including Regulation 854/2004 (2074/2005) [12] (Annex III Chap. II, C). 

Competent authorities are obliged to make decisions based upon on-going monitoring, and in the case of exceeding the standards for chemical hazards such as biotoxins, CAs are required to close production areas. Such closures effectively prohibit the harvesting of animals for human consumption, and CAs are then obliged to monitor compliance with these closures. With closures, the molluscs remain in the marine environment with view to depuration of toxin levels through normal biological filtration. Subsequent re-opening of production areas by CAs may only arise following two consecutive results below the regulatory limit that is derived from samples taken more than 48 h apart. 

In addition to this focus on primary phase of food production, CAs are obliged to establish a control system comprising laboratory tests throughout the bivalve mollusc food chain to verify the compliance of FBOs and products with regulatory requirements for hazards including marine biotoxins. Some products such as pectinidae and non-filter feeding gastropods may be harvested outside of classified production areas, and official controls are then required to verify compliance with health standards after harvest in fish auctions dispatch centres and processing establishments. 

### 2.5. Specific Regulatory Obligations for Marine Biotoxins: Food Business Operators

In addition to the various general obligations around hygiene, traceability, and food safety management, Operators involved in bivalve mollusc food chain are obliged by Regulation 853/2004 to incorporate specific systems into their food safety management systems, prescribed to explicitly manage the inherent microbial and biotoxin risks in this food-chain [1] (pp. 56–62). 

Primary food producers may only gather or harvest bivalve molluscs for which classification is required, from within classified production areas. They may not harvest from those areas that are unsuitable for health reasons. A salient specific regulatory requirement for this food chain pertains to the latter stage of the primary phase of this foodchain—movement of live animals following harvest—when normal generic traceability requirements are enhanced to oblige bivalve molluscs to be accompanied by a ‘registration document’ communicating various pieces of information such as health status of production area to the dispatch centre or processing establishment. Bivalve molluscs remain primary food products while accompanied by such registration documents, and may only be placed on the market for human consumption following handling or processing in at least one of the approved establishment types, thereby becoming subject to incumbent food safety management systems of those operations. In general terms, live bivalve molluscs accompanied by registration documents may be regarded as live animals not prepared for human consumption, whilst live bivalve molluscs or molluscan fishery products accompanied by documents containing an Identification Mark set out in Regulation 853/2004 pertaining to approved food establishments may be regarded as being on the market as food for human consumption. These foods may be either live bivalve molluscs coming from approved Dispatch Centres or molluscan fishery products coming from approved mollusc processing establishments.

Regulation 853/2004 sets out health standards that operators must ensure are reached by live bivalve molluscs when placed on the market for human consumption, stipulating that they should not exceed marine biotoxins in total quantities (measured in the whole body or any part edible separately) in excess of prescribed limits (Table 1). In the case of fishery products derived from molluscs, those animals must have been derived in accordance with the requirements for live bivalve molluscs, including marine biotoxin health standards.

### 2.6. Revision of Official Control Provisions

A fundamental reframing of the various official control provisions described is under progress through the enactment of Regulation (EU) 2017/625 on official controls and other activities performed to ensure the implementation of food and feed law and other rules (2017/625) [13]. This Regulation repeals and replaces various EU Regulations and Directives currently in force on official controls. Of direct relevance to the present review is the repeal of Regulations 882/2004 and 854/2004. Whilst some of the provisions of this new regulation around the role of European Union Reference laboratories (EURLs) come into effect in April 2018, and the bulk of 2017/625 provisions including those specific repeals come into effect in December 2019. 

The various articles of 2017/625 directly replace the various provisions of 882/2004; however, as regards the detailed provisions contained in the Annexes of current 854/2004 that set the specific obligation of CAs with regard to bivalve molluscs and fishery products, 2017/625 is purely an enabling framework regulation. Under the ‘commitology’ provisions contained in Articles 290 & 291 of the Treaty on the Functioning of the European Union (TEFU), this new regulation 2017/625 functions as primary legislation. It sets out broad principles and empowers the casting of secondary legislation to establish supporting detailed provisions by way of Delegated and Implementing Acts. Thus, the detailed official control requirements currently set out in Regulation 854/2004 will cease to exist at the end of 2019, and their direct replacements are, at the time of writing, under discussion with view toward enactment within necessary timeframes. This recasting of detailed official control provisions does not directly affect the operator provisions set out in regulations 852/2004 and 853/2004, but it is possible that some consequential changes may arise to ensure coherence with any modification to official controls.

## 3. Discussion

### 3.1. Official Control Infrastructure versus Operator Responsibility

As described above, the EU regulatory framework requires the development and maintenance of systems by Competent Authorities to classify production areas, monitor both phytoplankton in water and biotoxin levels in molluscs of those areas, and act where necessary to close and subsequently re-open those areas. Whilst Regulation 854/2004 explicitly allows CAs to take operator samples into account, this is ostensibly a significant dedicated official control infrastructure without which bivalve molluscs cannot be produced as food.

There are obvious strengths to an obligatory Official Control infrastructure, primarily in the assurance that arises from an agency of government acting in the public interest. Competent Authorities are effectively obliged to act as an informed independent gatekeeper to prevent unsafe product being harvested. It would be reasonable to regard an official control system as lacking the inherent conflicts that might exist in a system that is reliant on the checks of FBOs who might have to balance risks of rising toxins with commercial pressure to continue to place product on market. Furthermore, CAs will generally have the infrastructure to establish communal sampling programmes that cover production facilities shared by several operators—for example, within one classified production area—and to impose closures across various operators in those shared production areas in a harmonised way.

The current general EU food safety regulatory framework is the outcome of a fundamental reformulation of Food law according to the principals set out in the EU ‘White Paper on Food Safety’ [14]. A particular emphasis of that recasting was one of moving from official responsibility for gateway checks of end-product safety or suitability to operator bearing primary responsibility for food safety [14] (Art 14 & 17). The benefit of such an approach is the proactive engagement of the inherent complicity of FBOs in the food that they produce. It is not the CAs’ food product but the operators’ food product that is placed on the market. It is imminently more beneficial for the commercial viability of a food business operator that they ensure the safety of the product they place on the market, instead of risking long-term reputational loss in the face of short-term financial gain. Operators have on going first-hand involvement in the production checks and dispatch of every batch of food produced, whereas official controls exist on the basis of discrete risk-based, partial-presence interventions as opposed to continuous monitoring. On a practical basis, operators are involved in every harvest and dispatch of all molluscs and CAs are not.

To a certain extent, the comprehensive, detailed official control obligations that exist around bivalve molluscs might be somewhat at variance with that central tenet of FBO responsibility. In FBO responsibility systems, the primary role of the CA is verification of operator compliance with their obligations, not development of bespoke official food safety risk-management systems. Operators are explicitly obliged to consider the particular hazards of the food they produce and establish appropriate risk management strategies commensurate with their operation, while regulatory risk management of inherent food chain hazards has generally evolved away from imposition of dedicated official control systems layered on top of the general obligations. By way of context, the LBM food chain is not the alone in posing specific food safety risks. Other examples certainly exist whereby CAs are explicitly required to establish dedicated bespoke official control risk management systems in addition to both general and specific operator obligations, e.g., meat inspection at terrestrial animal slaughter or checks of dairy animals within the primary phase of that food chain. However, there are various examples of food safety hazards entering food chains in the pre-harvest phase, e.g., *Salmonella enteritidis* in laying poultry, in which EU regulations do not require official classification of terrestrial agricultural production geographic regions with consequent cessation of harvesting when concentrations exceed defined limits. 

A particular risk from overt reliance on dedicated official control infrastructure is the consequential perceived redirecting of operator responsibility for food safety onto the competent authority. Whist FBO sampling effort may frequently significantly contribute to official systems, CAs should maintain overall control of the system. In simple terms, the official control system involving weekly official sampling becomes the most dominant component of marine biotoxin risk management, both at that point of the food chain and, indeed, throughout the food-chain. It is increasingly normal for CAs to publish the results for individual classified production areas in the public domain, e.g., on the internet. This public-domain outcome is therefore seen to define the biotoxin safety for all molluscs derived from that area for that period, frequently constituting the dominant pillar of biotoxin risk management in the food safety management system for many of the operators handling those molluscs further along the food chain. The absence of closure of a production area is frequently interpreted as an absolute guarantee of adequate management of marine biotoxin risk in those molluscs.

Official control systems are designed for on-going monitoring of general health status of production area, not as components of individual FBO food safety management systems. Analogies of obligatory official control systems exist in other food chains, and by way of example the obligatory CA meat inspection systems in terrestrial slaughterhouses should not constitute the central component of the food safety management systems of those establishments, nor the meat cutting plants or meat processing establishments later in the food chain.

Overall, there is an inherent risk of dilution of FBO responsibility for food safety contrary to general provisions of EU regulatory framework that arises directly from the existence of high-profile CA systems deemed to be the defining arbiter of food safety as regards biotoxin risk.

### 3.2. Shared Responsibility across Holistic Food-Chain versus Individual Control Point

Monitoring during pre-harvest production and closures of production areas when necessary are obligatory components of the EU regulatory framework. The effective outcome is an official gateway through which molluscs must pass in order to become food. Not only does the system have to exist, but the sample has to comply in order for animals to pass from there to the next phase of the food chain. Passage through this gateway confers subsequent connotations of negligible biotoxin risk thereafter, and there is an inherent logic to this in the case of biotoxins that arise only during the pre-harvest phase. 

However, a further central tenet of the EU regulatory framework is that of a comprehensive integrated holistic food-chain approach to food safety, described in various terms such as ‘Farm to Fork’, ‘Stable to Table’, ‘Gate to Plate’, ‘Boat to Throat’, and various others [7,14]. This concept is one of shared responsibility in which every operator in the food-chain plays their respective part in managing food safety. Supported by meaningful traceability, this whole-chain approach should proactively engage all operators in their individual contribution to the shared collective responsibility for the safety of the ultimately consumed food product.

In the case of bivalve mollusc biotoxin risk management, instead of a shared cumulative responsibility by all operators, the various systems of checks and responsibility along the food chain frequently converge back to the monitoring programme preceding harvest. This may well be an individual sample for an extensive classified production area that covers numerous producers of different primary food products that go to different processing methods, contributing to even greater variation of final food products. An individual official programme sample becomes the risk assessment and risk management reference point for all harvested products, as such products are distributed throughout all subsequent food-chains. 

Within the holistic food-chain approach, the overall EU regulatory framework envisages additional checks by operators throughout the food-chain. Bivalve mollusc biotoxin risk management can therefore frequently align poorly with the concept of shared responsibility through the food-chain.

### 3.3. Offical Monitoring of Farmed Inshore Mollusc Production vs. Wild Offshore Capture Fisheries

A specific consideration as regards an official categorisation system for the health of the pre-harvest production phase is the applicability to the primary phase of molluscan wild fishery foodchain. A significant proportion of EU mollusc production comes from wild fisheries including capture systems at sea, both inshore and offshore, as well as raking and picking in foreshore and intertidal areas. A system of delineating, classifying, monitoring, and closing/opening production areas has a robust applicability for inshore areas with farmed non-motile animals. The extrapolation of such a system to wild motile animals captured in variable offshore locations is not necessarily straightforward or logical. In simple terms, neither the operators nor the CAs have as much knowledge or control of the pre-harvest phase of wild fisheries as might be the case for farmed mollusc production. Whilst anthropogenic factors constitute the dominant drivers of microbial safety in inshore waters, such considerations are generally irrelevant in offshore waters. By way of generality, the official control system of monitoring, closures, and reopening can, at most, be reactive to the commercial fisheries that are active in the case of wild offshore fisheries, as opposed to the proactive premise that is applicable to farmed production set out in the EU regulation for biotoxin monitoring.

### 3.4. Regulatory Reliance on Biotoxin Concentration in Raw/Live versus Comprehensive Food-Chain Approach

A further consideration is the manner in which the EU regulatory framework generally sets out maximal concentrations of biotoxins in live bivalve molluscs, measured in the whole animal or any part edible separately. Whilst some mollusc species, particularly oysters, may be consumed in the live entire raw state, other consumption patterns are also prevalent, e.g., consumption of cooked parts of animals or whole animals. The most common farmed mollusc species in the EU is mussels, and the most common wild-caught mollusc species is scallop. Mussels are frequently thermally processed (cooked), and scallops are frequently processed to remove inedible tissues such as shell or various body-parts and then cooked prior to consumption. Freezing and canning of molluscs is also a common part of the mollusc food-chain [6].

EU regulations require thermal processing such as cooking or canning of molluscs other than those meeting Class A microbial standards prior to placing them on the market for human consumption. Following cooking, the biotoxin concentration in cooked bivalve molluscs is approximately two-fold higher than the concentration in those molluscs if analysed when raw, due to various cooking effects including dehydration of mollusc tissue [15]. Removal of the hepatopancreas and digestive tract of LBMs with unacceptable biotoxin concentrations in the whole animal can produce edible parts of molluscs with safe biotoxin levels and is a useful biotoxin risk management strategy that is sometimes applicable for larger molluscs such as scallop [16]. 

The biotoxin limit applicable to live raw bivalve molluscs in EU legislation is overtly focused in its applicability to the matrix of live, and therefore, raw, fresh (unfrozen) and entire molluscs. For fishery products that comprise cooked or frozen entire molluscs, the regulations remain focused on compliance with the biotoxin limits applicable in the live molluscs from which those products were derived. 

Thus, for biotoxins, the general concept of holistic food chain controls cannot therefore be applied to substantial portions of the food chain when molluscs are not placed on the market as live entire animals, with resultant reliance by FBOs producing on the pre-harvest monitoring. The specific requirement of Regulation 854/2004 to establish a control system that comprises laboratory tests to verify FBO compliance with contaminant limits in end products of bivalve mollusc throughout food-chain cannot easily be applied with regard to biotoxin in fishery products derived from molluscs. In practical terms, the most meaningful checks are frequently those that verify the adequacy of traceability back to the primary phase of production—normally, the classified production area with acceptable biotoxin concentration.

### 3.5. Focus on Sampling and Analysis versus Process Control

A sample is designed to be a representative subset of a larger entity but is inherently an imperfect indicator of the status of that larger entity. Homogenous distributions are exceedingly rare in nature, and individual sample results have a confidence interval around which the non-sampled subunits in the population may be assumed to cluster. In the case of the biotoxin concentration of a population of molluscs in a classified production area, it is biologically normal to have a degree of variation amongst the individual animals. Regulation 854/2004 requires representative sampling points to be established in classified production area, with resultant implication of samples taken from this giving some indication of the central tendency of sample subunits in the population. In practical terms, a sample result of LBMs from an area gives useful and meaningful indication of the biotoxin concentration in animals from that area; however, no sampling system will ever absolutely guarantee that all animals in that area are within certain parameters, e.g., below the legal limit for biotoxins.

Sampling and analysis is generally set out in EU regulatory frameworks as a validation of the effectiveness of a food safety management system. That general framework does not envisage sampling onto-itself as the entirety, nor indeed the central component, of food safety management. In that context specific intent of the casting of the current EU regulatory framework was to move food safety risk management towards the concept of process management and away from product analysis as an index of safety [14]. Safety cannot be sampled into food, so biotoxin risk management would benefit from the widest possible spectrum of consideration, such as traceability and provenance assurance, assessing inherent risk at specific times of production year, hydrodynamic and mollusc physiology factors creating differential risk amongst the animals within a production area, and also intended market with likely portion size and ingestion quantity. Sampling to derive safety assurance based on not exceeding a legal limit might best be described as a blunt risk management tool.

### 3.6. Prospective Extrapolation vs. Retrospective Accumulation Outcome

The biotoxin concentration of LBMs is a reflection of the equilibrium that exists between the animal tissues and the contents of water that is filtered by the animal. At a simplistic level, ignoring any physiological sequestration by molluscs, when the toxin content of phytoplankton cells present in water is higher than that of the animal tissues, toxic concentration increases in the animal, and when the toxin content of phytoplankton cells present in water is lower than in the animal, concentration decreases in animal tissue. Raised biotoxin concentration in flesh of LBMs therefore indicates increased toxin content of phytoplankton cells in the water that was filtered in the immediate past by the mollusc.

A monitoring system that decides whether to assign open or closed status to an area for the period that begins immediately after sampling is one based upon forward-extrapolation of the applicability of the sample result. Whilst recent history of toxin accumulation in mollusc flesh has some correlation with future accumulation likelihood, the biological reality is that such samples are a much more accurate indication of recent history than a likely future. Some toxins, particularly water-soluble toxins, can accumulate and depurate very rapidly, and the ability of a sample to prospectively indicate future risk is particularly dubious.

On an individual result basis, it is, to a certain extent, reasonable to deduce a greater likelihood of both on-going immediate future toxin exposure and/or residual toxin retention in LBMs as future-extrapolation following an individual high result. On a trend-analysis of multiple samples from an individual area, the accumulation curve, plateau phase, and depuration phase of toxins that arise from harmful algal blooms can be recognised with reasonable confidence as increasing the accuracy of risk—based openings or closures based upon forward-extrapolation. Furthermore, the regulations also require monitoring of phytoplankton, which has arguably greater utility as an indicator of biotoxin risk in the future, particularly on the upward slope of an algal bloom. 

The sensitivity of prospective-extrapolation monitoring regimes to food risks is arguably weakest in scenarios in which one sample has substantially higher biotoxin concentration than previous sample, with an area becoming abruptly closed from the point of time beginning with the ‘high’ sample. That high result indicates increased toxin content in phytoplankton cells in the water and thence animals, arising at some stage following the ‘low’ sample, with every likelihood of food harvested later in that inter-sample period tending toward the higher concentration detected in the second sample. 

There are practical impediments to establishing monitoring programmes using samples as retrospective indicators of status—in particular, the potential for retrospective attribution of risk associated with food already on the market with consequent ongoing potential for recurrent withdrawals and recalls despite active compliance at the time of placing on the market. Such uncertainty could well create poor balance between regulatory impact and public health protection. A practical middle-ground might involve retaining stored, normally-untested ‘library samples’ for harvested batches to be analysed in order to provide greater confidence around the rising curve of biotoxin concentration and definitively, albeit retrospectively, identifying the time after which safe concentrations had been exceeded. In a manner more consistent with the precautionary principle, the re-opening of a production following an algal bloom can only arise in EU regulations following two compliant samples at least 48 h apart to show depuration to safe concentrations had already occurred at some time before the first of the two samples.

### 3.7. Risk-Based Official Controls, vs. Monitoring & Surveillance

Competent Authorities are obliged to have a comprehensive monitoring programme in place for phytoplankton and biotoxins in water and flesh, respectively. With a baseline expectation of weekly toxin monitoring in production areas with active harvesting, the cumulative dataset generated across the EU Member States is comprehensive. Regulation 882/204 defines monitoring as a planned sequence of observations or measurements with view to obtaining an overview of the state of compliance [8] (Article 2.8). However, Regulation 854/2004 explicitly requires monitoring of phytoplankton and biotoxins to be risk-based with enhanced sampling when risk is perceived to be higher and to have interventionist outcomes including area closures by Competent Authorities. Such programmes are therefore designed to contribute to public health risk management as opposed to understanding prevalence or distribution of toxins. These programmes are primarily components of control programmes as opposed to epidemiological tools.

There are, however, relevant analogies in comparable EU regulations around monitoring of other chemical substances and residues in food (Directive 96/23/EC) [17], and microbiological zoonoses and zoonotic agents (Directive 2003/99/EC) [18]. In those cases, such regulatory monitoring results in EU-level reporting of outcomes based on data produced by Member State monitoring programmes. Whilst such collated data would need to be interpreted in the context of the underlying risk basis of sample selection, these reports usefully add to the value of such substantial official monitoring undertaking. In particular, for biotoxin official control monitoring such cumulative reporting might usefully contribute to better understanding of time-trends in prevalence and geographical or temporal distribution of toxins. 

## 4. Conclusions

### 4.1. Overall Adequacy versus Burden of Compliance

Perfect or ideal food safety risk management systems do not exist; all such systems represent a balance between negative impact such as costs on food production, burden on state services, or impingement of consumer choice and food security, and the positive impact on public health and industry reputation. The truest index of adequacy food safety of a risk management system is the achievement of the defined Appropriate Level of Protection (ALOP) in a population. This is might be expressed as a public health goal perhaps incidence of illness in total population or consumers of a food product. It is then possible to derive the Food Safety Objective (FSO), which correlates with that goal, in the current context the maximal permissible concentration [19]. However, ALOPs are exceedingly rarely defined in food safety due to a comparative intolerance for overt acknowledgement of anything greater than zero risk. Moreover, for many foodborne illnesses, the incidence of illness and the relative attribution to particular food-chains are relatively poorly understood.

The overall adequacy of a risk management system is therefore more frequently assessed on more subjective terms of perceived public health benefits in comparison to the burden compliance. It is it is reasonable to describe a system that requires weekly official water and molluscs sampling of every classified production area, and has the subsequent potential for abrupt complete cessation of food production on any week, as bringing a relatively large burden of compliance both for CAs and FBOs. As regards public health, the data available reflect the situation with the system in place, so they are more of an index of the residual risk not dealt with by the system, or in all likelihood are either in intentional or accidental non-compliance with the system. Those available data indicate that an ongoing contribution of bivalve molluscs to foodborne illness, with the food-group ‘molluscs crustaceans and products thereof’ implicated in 9.5%, 8.2%, and 7.3% of strong-evidence food-borne outbreaks in the EU in 2015 [2], 2014 [20], and 2013 [21], respectively. Data indicate a variable contribution of biotoxins to that burden of illness, generally secondary to Norovirus as regards number of outbreaks associated with molluscs and also lower than histamine in the case of fishery products [21] (p. 266). 

One tangential but arguably useful index of the perceived adequacy of the EU regulatory current system as regards consumer protection is the manner in which more recent biotoxin toxicity assessments that recommend substantial reduction in acceptable concentration of most of the regulated compounds [3] have not to date been deemed necessary to be implemented in EU legislation.

### 4.2. The EU Biotoxin System in Changing World

Regulatory risk management regimens are the outcomes of democratic process informed by scientific understanding of risk and various societal drivers including regulatory impact assessment. In the case of biotoxins as a foodborne hazard, understanding around their toxicity, analysis, prevalence, and the epidemiology of resultant human disease, as well as characterisation of consumption patterns of molluscs and their products, as well as understanding sociological drivers of compliance with food safety obligations, have all developed since the casting of these regulations. Societal willingness to support overt reliance on regulatory infrastructure as a risk management strategy, as a supplement to operator responsibility, also continues to evolve. The food chain itself, including production approaches, complexity of distribution chains, and consumption patterns also change over time. The actual epidemiology of marine biotoxins is also evolving with hitherto exotic toxins emerging in EU [22].

The EU food regulations are devised around a pre-supposition of on-going likelihood of problematic concentrations of marine biotoxins that arise in bivalve molluscs during the primary phase of this food-chain. The system essentially seeks to identify when such events occur and plans to be reactive to such events. The framework brings an overt reliance on official control systems in that primary phase prior to the harvest of these animals for food. The key risk assessment tool is the sampling and analysis of phytoplankton and biotoxins, and the key risk management tool is the prohibition of harvesting of live animals to become food when marine biotoxins health standards are exceeded. This approach brings both advantages such as robust independent scrutiny and systematic monitoring infrastructure, and disadvantages such as reduced operator responsibility for food safety and substantial extrapolation from individual sample data points. Systems involving more general alignment with the central tenets of FBO responsibility and holistic food chain process controls may merit on-going consideration in regulatory risk management.

## Figures and Tables

**Table 1:** Summary of Biotoxin Health standards prescribed in Regulation 853/2004 [1] for Live Bivalve Molluscs, measured in the whole body or any part edible separately.

Biotoxin Group	Abbreviation	Maximum Permissible Quantity
Paralytic Shellfish Poison	PSP	800 micrograms per kilogram
Amnesic Shellfish Poison	ASP	20 milligrams of domoic acid per kilogram
Okadaic acid, Dinophysistoxins and Pectenotoxins together	DSP	160 micrograms of okadaic acid equivalents per kilogram
Yessotoxins	YTX	3.75 milligrams of yessotoxin equivalent per kilogram
Azaspiracid	AZP	160 micrograms of azaspiracid equivalents per kilogram

## Disclaimer

The summaries of legislative requirements are provided for illustrative purposes only and are not exhaustive; therefore, interested parties should refer to legislation for complete details of legal obligations. The contents and appraisals represent the contemporaneous views of the author based upon a specialism in Veterinary Public Health, experience in food safety regulation, and a specific role including regulatory risk management of marine biotoxins in bivalve molluscs. The contents and opinions should not be regarded as representing the views of any individual Competent Authority, government administration, nor Member State.
